# Plasma specific miRNAs as predictive biomarkers for diagnosis and prognosis of glioma

**DOI:** 10.1186/1756-9966-31-97

**Published:** 2012-11-22

**Authors:** Qiong Wang, Pengcun Li, Ailin Li, Wei Jiang, Hong Wang, Jinhuan Wang, Keliang Xie

**Affiliations:** 1 Tianjin Neurosurgery Institute, Tianjin Huanhu Hospital, 122 Qixiangtai Street, Tianjin, 300060, P.R. China; 2 The Graduate School, Tianjin Medical University, 22 Qixiangtai Street, Tianjin, 300070, P. R. China; 3 Department of Anesthesiology, General Hospital of Tianjin Medical University, Tianjin, 300052, P. R. China

**Keywords:** Glioma, MicroRNA, Plasma, Biomarker

## Abstract

**Objective:**

Glioblastoma multiforme (GBM) is a highly malignant brain tumor with a poor prognosis. MicroRNAs (miRNAs) are a class of small non-coding RNAs, approximately 21–25 nucleotides in length. Recently, some researchers have demonstrated that plasma miRNAs are sensitive and specific biomarkers of various cancers. The primary aim of the study is to investigate whether miRNAs present in the plasma of GBM patients can be used as diagnostic biomarkers and are associated with glioma classification and clinical treatment.

**Materials and Methods:**

Plasma samples were attained by venipuncture from 50 patients and 10 healthy donors. Plasma levels of miRNAs were determined by real-time quantitative polymerase chain reaction.

**Results:**

The plasma levels of miR-21, miR-128 and miR-342-3p were significantly altered in GBM patients compared to normal controls and could discriminate glioma from healthy controls with high specificity and sensitivity. However, these three miRNAs were not significantly changed in patients with other brain tumors such as meningioma or pituitary adenoma. Furthermore, the plasma levels of these three miRNAs in GBM patients treated by operation and chemo-radiation almost revived to normal levels. Finally, we also demonstrated that miR-128 and miR-342-3p were positively correlated with histopathological grades of glioma.

**Conclusions:**

These findings suggest that plasma specific miRNAs have potential use as novel biomarkers of glioma and may be useful in clinical management for glioma patients.

## Background

Glioblastoma multiforme (GBM, a grade IV glioma) is a primary brain tumor that is highly malignant, and the patients diagnosed with GBM remain poor prognosis despite implementation of intensive therapeutic strategies and clinical efforts. To date, the diagnosis of GBM before clinical treatment is mainly by computer tomography (CT) and nuclear magnetic resonance imaging (MRI). However, they are expensive and difficult to spread. Therefore, it is an urgent need to find new approaches to early diagnose GBM and monitor disease progress.

MicroRNAs (miRNAs) are a large class of small non-coding RNAs that regulate gene expression at the post-transcriptional level [1]. MiRNAs are thought to regulate expression of more than 30% of messenger RNAs and play a viral role in many physiological and pathological processes such as cellular proliferation, differentiation, apoptosis, carcinogenesis, cancer cell invasion [2-4]. Additionally, more and more researchers also found that circulating miRNAs of plasma or serum (extracellular miRNAs) could be used as potential biomarkers for detection, identification, and classification of cancers and other diseases because (1) miRNAs expression is specific in different tissues [5], (2) the expression levels of miRNAs are changed in cancers or other diseases [6,7], (3) miRNAs of plasma or serum is a remarkably stable form and can be detected in plasma [8].

Baraniskin *et al.* found that miRNAs in cerebrospinal fluid (CSF) could be referred to as biomarkers for diagnosis of glioma [9]. However, it is difficult to attain CSF. In addition, Roth *et al.* also demonstrated that specific miRNAs in peripheral blood also may be suitable biomarkers for GBM [10]. But miRNAs of blood cells may interfere with the accuracy of the results. Thus, miRNAs in plasma or serum could be developed as a novel class of blood-based biomarker to diagnose and monitor glioma.

Up to now, previous studies have documented that a number of miRNAs, including miR-21, miR-128, miR-15b, miR-221/miR-222, miR-181a/b/c and miR-342-3p, were dysregulated in glioma tissue [10-14]. These miRNAs play a vital role in anti-apoptosis, proliferation, invasion, and angiogenesis of glioma cells. In this present study, therefore, these miRNAs were chosen and detected in plasma samples of glioma patients as well as healthy controls. The primary aim of the study was to investigate whether GBM-associated miRNAs in plasma could be used as diagnostic biomarker of glioma patients, and whether these miRNAs significantly altered could reflect the glioma classification, stage of disease and effect of clinical treatment.

## Methods

### Ethics statement

The study was approved by Research Ethics Committee of Tianjin Huanhu Hospital. All clinical samples described here were gained from patients who had given informed consent and stored in the hospital database.

### Clinical samples

Plasma samples for miRNAs detection were collected from patients with pathologically confirmed glioma (grade II-IV) (n = 30), pituitary adenoma (n = 10) and meningioma (n = 10) before surgery at Department of Neurosurgery, Tianjin Huanhu Hospital from January, 2011 to April, 2012. In addition, plasma samples of GBM patients (n = 10) were obtained in preoperation, two weeks after surgery and a month after X-ray radiotherapy and temozolomide chemotherapy, respectively. The detailed characteristics of these patients are shown in Table 1. Plasma samples from healthy donors (n = 10) were obtained. The blood samples were obtained and centrifuged for 10 min at 1,500 g within 2 h after collection, and the supernatant was removed to RNase-free tubes and further centrifuged for 10 min at 12,000 g and 4°C to remove cells and debris. Plasma was stored at −80°C until further processing.

**Table 1:** Characteristic of brain tumors patients

**Brain tumors**					
**Tumor types**	**Glioma**			**Meningioma**	**Hypophysoma**
	**Grade II**	**Grade III**	**Grade IV**		
Sample Size	n=10	n=10	n=10	n=10	n=10
Average Age	47.9	45.9	51.3	46.1	49.2
Range	25-71	25-72	27-75	18-60	35-73
Sex					
Male	5	4	5	4	4
Female	5	6	5	6	6

### MiRNAs isolation and quantitative reverse-transcriptase polymerase chain reaction (qRT-PCR)

MiRNAs were extracted from 400 μL of plasma using the miRcute miRNA isolation kit (Tiangen biotech C, LTD. Beijing) according to the manufacturer’s protocol. Briefly, 400 μL Lysis Solution and 200 fmol mmu-miR-295 mimics (Qiagen, USA) were added into 400 μL plasma and incubated for 5 min and centrifuged for 10 min at room temperature. The supernatant was removed and added 200 μL chloroform, and then the mixture was centrifuged at 12,000 g for 15 min. Aqueous phase was transferred to an absorption column in the miRNA extraction kit. MiRNAs were absorbed in the column and then solution C was added to remove the protein, the waste solution was removed by centrifuge. The column was washed with wash solution in the kit for twice, and finally the miRNAs were dissolved in 20 μL RNase-free water. Subsequently, the miRNA samples were stored at −80°C. MiRNAs was quantified using the NanoDrop 1000 (NanoDrop, Wilmington, DE).

A SYBR Green-based quantitative RT-PCR assay was performed in order to quantify miRNAs in isolated plasma samples. For each target, 2 μg of plasma miRNAs for each subjects was reversely transcribed in 10 μL reaction system containing: 1 μL miScript Reverse Transcriptase Mix, 4 μL 5×miScript RT Buffer and 0.5 μL (100 pmol/μL) primer (sequences shown in Table 2), and the mixture was added with RNase-free water to 10 μL volume. The mixture was incubated at 65°C for 10 min, 42°C for 60 min, followed by 70°C for 10 min. Real-time PCR was employed with a SYBR Premix Ex Taq (TaKaRa, Dalian, China), all specific primers for miRNAs were synthesized by AuGCT DNA-SYN Biotechnology (Beijing, China) (sequences shown in Table 2). Real-time PCR reactions were carried out in a total volume of 20 μL reaction mixture containing: 1 μL of RT product mixed with 0.5 μL (10 pmol/μL) forward and reverse primer respectively, 10 μL of SYBR Premix Ex Taq and 8 μL of water. The procedure for PCR was 94°C for 3 min; 94°C for 30 s, 56°C for 30 s, 72°C for 50 s, 45 cycles, 72°C for 10 min. All reactions including controls were performed in triplicate using ABI 7500 PCR system (ABI, USA) and was normalized by spiked-in mmu-miR-295 expression for plasma (Previous research has confirmed mmu-miR-295 is absent in normal human serum [15]).

**Table 2:** The sequence of synthetic primers of PCR, RT and single-stranded miRNAs

**miRNA ID**	**Primer and miRNA**	**sequence**
	RT	GTCGTATCCAGTGCAGGGTCCGAG
		GTGCACTGGATACGACTCAACATC
hsa-miRNA-21	Forward primer	TGCGGTAGCTTATCAGACTGATG
	Reverse primer	CCAGTGCAGGGTCCGAGGT
	RT	GTCGTATCCAGTGCAGGGTCCGAG
		GTGCACTGGATACGACAAAGAGAC
hsa-miRNA-128	Forward primer	TGCGGTCACAGTGAACCGGTCTC
	Reverse primer	CCAGTGCAGGGTCCGAGGT
	RT	GTCGTATCCAGTGCAGGGTCCGAG
		GTGCACTGGATACGACACGGGTG
hsa-miRNA-342-3P	Forward primer	TGCGGTCTCACACAGAAATCGCAC
	Reverse primer	CCAGTGCAGGGTCCGAGGT
	RT	GTCGTATCCAGTGCAGGGTCCGAG
		GTGCACTGGATACGACACTCACC
hsa-miRNA-181a	Forward primer	TGCGGAACATTCAACGCTGTCGG
	Reverse primer	CCAGTGCAGGGTCCGAGGT
	RT	GTCGTATCCAGTGCAGGGTCCGAG
		GTGCACTGGATACGACACCCACC
hsa-miRNA-181b	Forward primer	TGCGGAACATTCATTGCTGTC
	Reverse primer	CCAGTGCAGGGTCCGAGGT
	RT	GTCGTATCCAGTGCAGGGTCCGAG
		GTGCACTGGATACGACACTCACC
hsa-miRNA-181C	Forward primer	TGCGGAACATTCAACCTGTCGG
	Reverse primer	CCAGTGCAGGGTCCGAGGT
	RT	GTCGTATCCAGTGCAGGGTCCGAG
		GTGCACTGGATACGACTGTAAAC
hsa-miRNA-15b	Forward primer	TGCGGTAGCAGCACATCATGGTTTAC
	Reverse primer	CCAGTGCAGGGTCCGAGGT
	RT	GTCGTATCCAGTGCAGGGTCCGAG
		GTGCACTGGATACGACGAAACC
hsa-miRNA-221	Forward primer	TGCGGAGCTACATTGTCTGCTGG
	Reverse primer	CCAGTGCAGGGTCCGAGGT
	RT	GTCGTATCCAGTGCAGGGTCCGAG
		GTGCACTGGATACGACACCCAG
hsa-miRNA-222	Forward primer	TGCGGAGCTACATCTGGCTACTG
	Reverse primer	CCAGTGCAGGGTCCGAGGT
	RT	GTCGTATCCAGTGCAGGGTCCGAG
		GTGCACTGGATACGACAGACTCA
mmu-miRNA-295	Forward primer	TGCGGAAAGTGCTACTACTTTTG
	Reverse primer	CCAGTGCAGGGTCCGAGGT
mmu-miRNA-295	RNA	AAAGUGCUACUACUUUUGAGUCU

### Statistical analysis

The statistical analysis was performed by *SPSS* version 13.0. The Mann–Whitney test was used to assess the differences between the healthy group and GBM patients. A *P* value of less than 0.05 was considered statistically significant. The Bonferroni correction was applied for multiple comparisons for between normal control group, glioma II, III and IV grade, or different brain tumors, *P-*values are significant at the 0.008 level adjusted for multiple comparisons with Bonferroni correction. The relative levels of microRNA were quantified using the 2^-△△Ct^ method. Receiver operating characteristic (ROC) curves were generated to assess the power of each miRNA to distinguish GBM patients from healthy group.

## Results

### The predictive value of plasma miR-21, miR-128 and miR-342-3p in GBM patients

**Figure 1:**
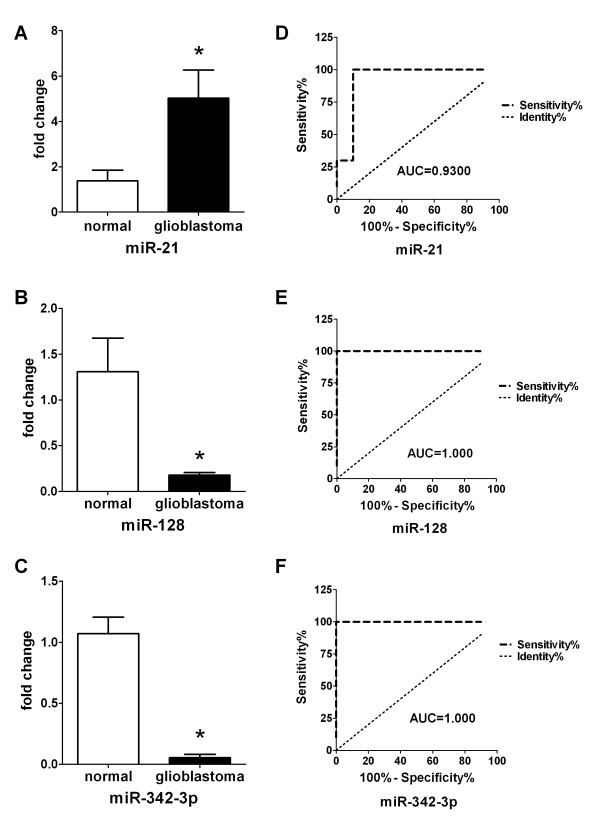
**Relative expression levels of miR-21, miR-128 and miR-342-3p in plasma from healthy controls and GBM patients, ROC curve analysis based on expression of each miRNA in plasma.(A, B, C)** Expression levels of the miR-21, miR-128 and miR-342-3p are normalized to mmu-miR-295 and analyzed using 2^-△△Ct^ method. Statistically significant differences were determined using the Mann–Whitney U test. Plasma levels of miR-21 are significantly higher in GBM samples than in control samples (*P* < 0.001), and levels of miR-128 and miR-342-3p are significantly lower in GBM samples than in control samples (*P* < 0.001). **(B)** The AUC for miR-21 was 0.9300 (95% CI: 0.7940-1.066) with 90.0% sensitivity and 100% specificity. **(D,F)** The AUC for miR-128 or miR-342-3p was 1.000 (95% CI: 1.000 – 1.000) with 90.0% sensitivity and 100% specificity.

**Figure 2:**
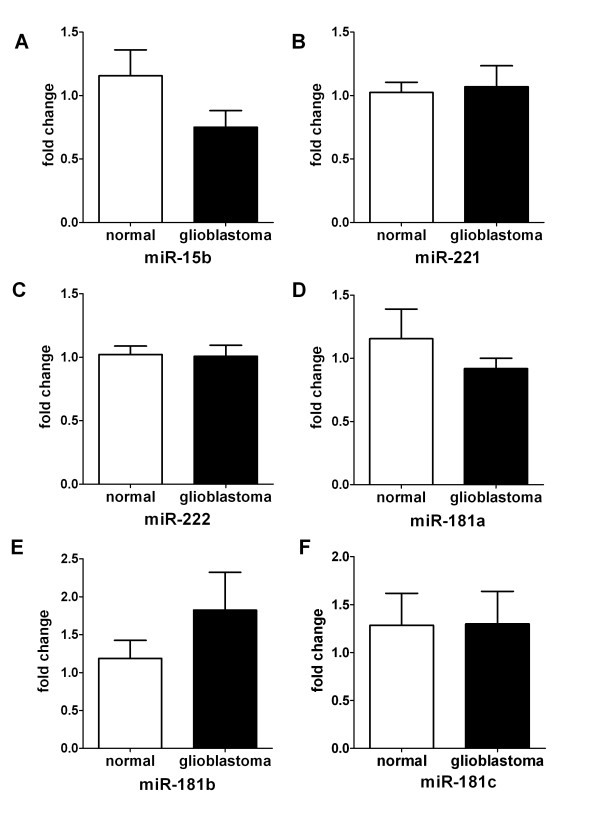
**Expression levels miR-15b, miR-221/222, miR-181a/d/c levels in plasma of healthy controls and GBM patients.** All these miRNAs are normalized to mmu-miR-295 and analyzed using 2^-△△Ct^ method. Statistically significant differences were determined using the Mann–Whitney U test. There was no significant difference between controls and GBM patients (*P* > 0.05).

We obtained 10 blood samples from patients with a histopathologically confirmed diagnosis of GBM and 10 healthy volunteers with a matched distribution of age and sex served as controls. In our studies, nine miRNAs (Table 3: miR-21, miR-128, miR-15b, miR-221, miR-222, miR-342-3p, miR-181a, miR-181b and miR-181c) were selected to examine their potential to serve as biomarkers for GBM. We analyzed whether these candidate miRNAs could serve as circulating markers by comparing their plasma levels between GBM patients and normal controls by real-time PCR. The results showed that miR-21 expression was significantly higher in the GBM group than in the control group (*P* < 0.001, Figure 1A), while miR-128 and miR-342-3p were remarkably lower in the GBM group (*P* < 0.001, Figure 1C and E). To determine whether the three plasma miRNAs could specifically and sensitively discriminate GBM from healthy controls, ROC curves were constructed. The ROC curves analysis showed that at the optimal cut-off, plasma miR-21 had a 90*%* sensitivity and a 100% specificity and the area under the ROC curve (AUC) was 0.9300 [95% confidence interval (CI): 0.7940-1.066)] (Figure 1B); miR-128 and miR-342-3p had a 90% sensitivity and a 100% specificity and AUC was 1.000 (95% CI: 1.000-1.000), respectively (Figure 1D and F). But plasma levels of miR-15b, miR-221, miR-222 and miR-181a/b/c did not show significant difference between controls and GBM patients (*P* > 0.05) (Figure 2A, B, C, D, E and F).

**Table 3:** Candidate miRNAs for investigation in the plasma of GBM

**miRNA**	**Previous association with Glioblastoma**
miR-21	High levels of miR-21 were first reported in glioblastoma
	tumors and cell lines compared to normal
	brain tissue [11,12].
miR-15b	Down-regulated in glioblastoma tissue compared to
	normal brain tissue [14]
miR-222/221	Increased expression in glioblastoma tissue compared to
	normal brain tissue [13]
miR-128	Down-regulated in glioblastoma tissue compared to
	normal brain tissue [13]
miR-181a/b/c	Down-regulated in glioblastoma tissue compared to
	normal brain tissue [13]
miR-342-3p	Expression level decreased in blood of the glioblastoma
	patients compared to th heathy donors [10]

### Association of the plasma levels of miR-21, miR-128 and miR-342-3p with histopathological grade of glioma

**Figure 3:**
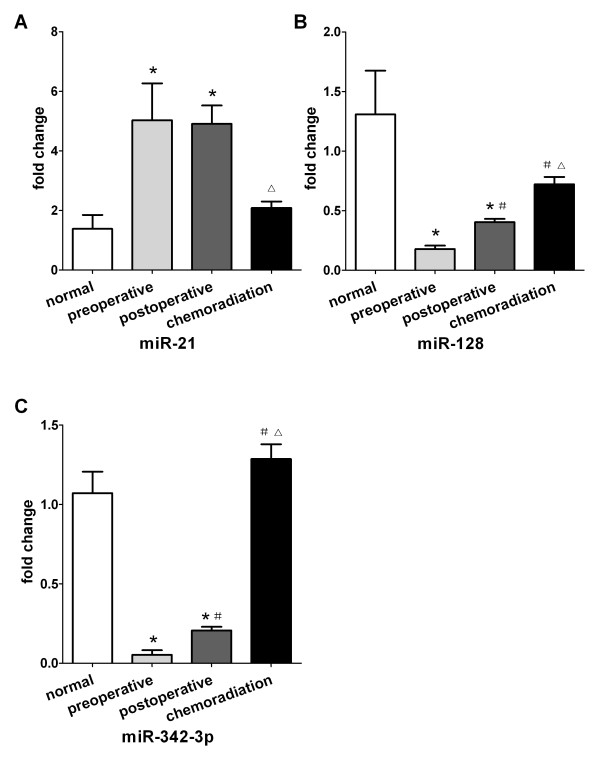
**The relationship between the plasma levels of miR-21, miR-128 and miR-342-3p and classification of glioma. (A)** Levels of miR-21 were up-regulated in grade II in comparison with control cohorts and much higher in grade III cohorts than in grade II cohorts, however there were no significant difference between glioblastoma patients (grade IV) and grade III cohorts or grade II cohorts. **(B)** Levels of miR-128 were significantly lower in grade II cohorts than in normal cohorts, much lower in grade III cohorts and in glioblastoma patients than in grade II cohorts (*P* < 0.001), there were no significant difference between glioblastoma patients and grade III cohorts. **(C)** Levels of miR-342-3p were significant difference among all formation. ^*^P<0.008 in comparison with normal, ^#^*P* < 0.008 in comparison with glioma II, ^△^*P* < 0.008 in comparison with glioma III.

In order to further explore the relationship between the plasma levels of miR-21, miR-128 and miR-342-3p and histopathological grade of glioma, we collected plasma samples from a group of normal cohorts (n =10), grade II (n = 10), grade III (n = 10) and GBM patients (grade IV) (n = 10) and detected the levels of miR-21, miR-128 and miR-342-3p using real-time PCR. Plasma level of miR-21 was significantly higher in grade II, grade III and GBM samples than in control samples (*P* < 0.001 Figure 3A), but we failed to find a relationship between its expression and clinical grades of glioma. Our data also showed a much lower level of miR-128 in high grades glioma (grade IV and III) than low grades glioma (grade II) (Figure 3B, *P* < 0.008); however, no difference was found between grade III and grade IV (Figure 3B, *P* > 0.008). There are significant difference in expression levels of miR-342-3p between grade II, III and IV (Figure 3C, *P* < 0.008). Plasma level of miR-342-3p was notably decreased in glioma with ascending tumor grades.

### Changes of miR-21, miR-128 and miR-342-3p levels in plasma samples of GBM patients after operation and chemo-radiation

**Figure 4:**
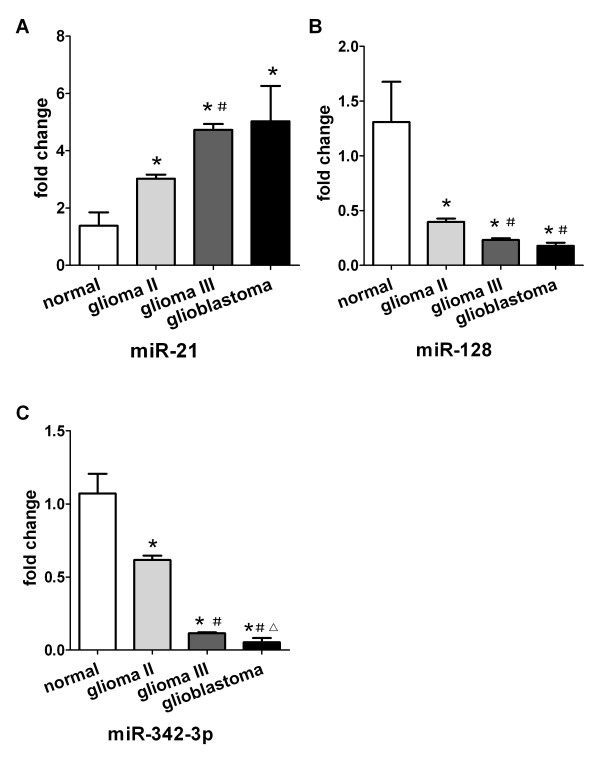
**The miR-21, miR-128 and miR-342-3p expression in normal control (n = 10), preoperation (n =10), postoperation (n = 10) and chemo-radiation (n = 10) plasma samples. (A)** Plasma levels of miR-21 are not significantly different between preoperative and postoperative patients, but levels of miR-21 are significantly lower in chemo-radiation cohorts. **(B)** and **(C)** Levels of miR-128 and miR-342-3p showed significant difference between cohorts of preoperation and postoperation and chemo-radiation. ^*^P < 0.008 in comparison with normal, ^#^*P* < 0.008 in comparison with preoperation, ^△^*P* < 0.008 in comparison with postoperation.

We chose 10 GBM patients and collected their plasma in preoperation, postoperation and after chemo-radiation. We found that the levels of miR-21 did not show significant difference between cohorts of preoperation and postoperation (*P* = 0.393), however, the levels of miR-21 was observably reduced after chemo-radiation (*P* < 0.001, Figure 4A). Furthermore, our data also revealed that the levels of miR-128 and miR-342-3p were markedly distinct between cohorts of preoperation, postoperation and chemo-radiation (*P* < 0.001, Figure 4B and C). After patients were treated by operation and chemo-radiation, the levels of miR-21, miR-128 and miR-342-3p revived to normal levels and did not differ significantly between chemo-radiation cohort and controls (*P* > 0.008).

### The expression levels of plasma miR-21,miR-128 and miR-342-3p specifically correlated with glioma

**Figure 5:**
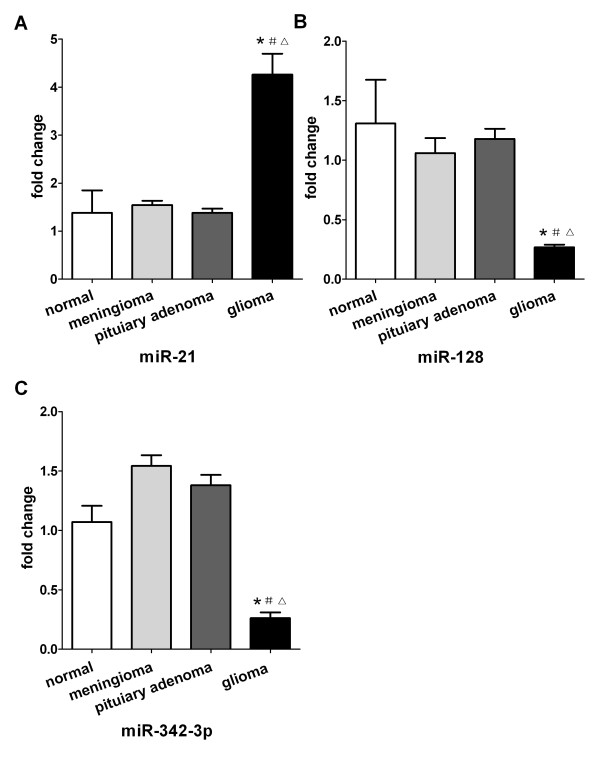
**Plasma levels of miR-21, miR-128 and miR-342-3p in normal cohorts, meningioma cohorts, pituitary adenoma cohorts and glioma cohorts. (A)** Plasma levels of miR-21 are significantly increased in glioma samples compared to control samples, **(B)** and **(C)** levels of miR-128 and miR-342-3p are markedly reduced in glioma samples compared to control samples. But there was no significant difference between controls and meningioma patients or pituitary adenoma patients (*P* > 0.05). ^*^P < 0.008 in comparison with normal, ^#^*P* < 0.008 in comparison with meningioma, ^△^*P* < 0.008 in comparison with pituitary adenoma.

To evaluate weather levels of plasma miR-21, miR-128 and miR-342-3p are specifically correlated with glioma, we selected a group of normal cohorts (n = 10), meningioma cohorts (n = 10), pituitary adenoma cohorts (n = 10) and glioma cohorts (n = 30). We found that plasma levels of miR-21 were significantly higher in glioma samples than in normal control samples (*P* < 0.001, Figure 5A), and levels of miR-128 and miR-342-3p were significantly lower in glioma samples than in control samples (*P* < 0.001, Figure 5B). In addition, there was no significant difference between controls and meningioma patients or pituitary tumor patients (*P* > 0.008, Figure 5C). The data suggest that the three miRNAs are specifically associated with glioma.

## Discussion

In the study, our results showed that miR-21 was up-regulated in plasma samples of human glioma tumors compared to healthy controls, whereas miR-128 and miR-342-3p were down-regulated. ROC analysis demonstrated the sensitivity and specificity of miR-21, miR-128 and miR-342-3p for GBM diagnosis. In order to further indentify the relationship between plasma level of the three miRNAs and classification and treatment effect of glioma, we next performed statistical analysis of our miRNAs expression data. There was a significant difference in plasma levels of miR-128 between the earlier stages (grade II) and the later subgroups (grade III and IV). Plasma level of miR-342-3p was notably decreased in glioma with ascending tumor grades. Expression levels of three miRNAs in plasma samples of patients treated reached levels comparable with control subjects. Additionally, the three miRNAs can specifically discriminate glioma from other brain tumor such as pituitary adenoma and meningioma.

MiRNAs were firstly discovered in 1993 when Lee *et al*. studied regulation of developmental timing in *Caenorhabditis* and reported a small RNA, lineage- definicient-4 (lin-4) [16]. To date, more than 1 000 miRNAs in human have been discovered according to miRBase sequence Database Release 14 (http://www.mirbase.org/). MiRNAs represent approximately 1% of the eukaryotic transcriptome. They play key regulatory roles in a diverse range of pathway, including tumorigenesis and progression of cancer. Furthermore, variation of specific miRNAs in plasma offers the potential for detection, subtype and prognosis determination.

MiR-21 level is markedly elevated in human GBM tumor tissues [11-13]. It targets multiple components and plays an anti-apoptotic function in GBM. We found that miR-21 is significant higher in plasma of GBM patients than in controls, which is consistent with the finding of miR-21 with significant levels in CSF sample and tissue from patients with glioma [9,11]. Furthermore, although circulating miR-21 is reduced in postoperation compared to preoperation, no significant difference existed. MiR-21 is observably decreased after further treatment with chemo-radiaton. Thus, these data suggest a possible association between miR-21 and treatment effect.

The expression level of brain-enriched miRNA-128 in glioma tissues is inversely correlated with tumor grade and function as a tumor suppressor [17]. Similarly, we found that expression level of miR-128 in plasma of GBM patients was also decreased and negatively relevant to high and low grade glioma, just same as the tendency reflected in the test results of glioma tissues. But another research reported that miR-128 was up-regulated in peripheral blood of GBM patients [10]. The reason may be that miRNAs contained blood cells cause the difference. Our data also revealed that miR-128 is up-regulated after glioma patients were treated, so miR-128 may be associated with curative effect.

To date, little is known whether miR-342-3p is dysregulated in glioma tissues and has an effect on glioma development. Roth *et al*. reported that miR-342-3p was down-regulated in peripheral blood of GBM patients [10]. In the present study, our results also showed that the expression level of miR-342-3p is reduced in the plasma of glioma patients and also inversely correlated with glioma grade. In addition, we assessed the expression of miR-342-3p by real-time PCR in the group of patients who had been treated by operation and chemo-radiation. miR-342-3p is significantly increased and there are no differences between normal, control plasma and plasma sampling received therapies. All these results reveal that plasma-derived miR-342-3p may be a suitable biomarker which can function as diagnosis, classification and therapeutic effect.

The mechanism of origin of extracellular miRNAs remains to be fully elucidated. Some researchers have demonstrated that miRNAs in plasma are released from cells in membrane-bound vesicles which are named microvesicles (exosomes). These exosomes come from multivesicular bodies and are released by exocytosis and also can be shed by outward budding of the plasma membrane [18-21]. These early reports are confirmed by which cultured cells release exosomes containing miRNAs [22-24]. Similarly, one study has also demonstrated that microvesicles (exosomes) containing miRNAs are released from glioblastoma cells and the size of them is from 50 to 500 nm [25]. The other two researches have revealed most of the extracellular miRNAs is bound to protein and form complexes rather than vesicles [26,27]. Comprehensive previous researches, we preliminarily speculate that miRNAs in the plasma of patients with glioma derive from glioma cells because (1) blood brain barrier (BBB) is partly destroyed in patients with glioma; (2) exosomes or complexes may be through the BBB by unknown mechanisms. It is necessary to further investigate if microvesicles encapsulation is the only mechanism for miRNAs in plasma with glioma or if other potentially more predominant mechanisms exist. One interesting point we observed in our study and other studies is that the expression level of some miRNAs is different in different body fluids. For example, our results found that miR-15b in plasma doesn’t dysregulate, but another study has indicated that it is significantly increased in CSF from patients with glioma compared to samples from control patients [9]. Because BBB exists, it is necessary to systematically explore the origin of plasma miRNAs of glioma patients and find the relationship between miRNAs of tumor cells and that of plasma.

In summary, our results demonstrate cell-free miR-21, miR-128 and miR-342-3p of plasma are specificity and sensitivity for diagnosis of GBM, suggesting that these miRNAs may be used as non-invasive biomarkers in GBM. Moreover, our data also find that particular miRNAs have a strong correlation with classification and clinical course and aid in therapeutic decisions for glioma patients through detecting plasma.

## Abbreviations

GBM: Glioblastoma multiforme; miRNA: MicroRNA; CSF: Cerebrospinal fluid; RT-PCR: Reverse-transcriptase polymerase chain reaction; ROC: Receiver operating characteristic; AUC: The area under the ROC curve; BBB: Blood brain barrier.

## Competing interests

The authors have declared that no competing interests exist.

## Authors' contributions

Conceived and designed the experiments: Jinhuan Wang, Conducted the experiments: Pengcun Li and Ailin Li, Analyzed the data and prepared the manuscript:Qiong Wang and Keliang Xie, Collected plasma samples: Wei Jiang and Hong Wang. All authors read and approved the final manuscript.
